# Public Health-Relevant Factors Associated with Gestational Diabetes Mellitus and Obstetric Outcomes: A Case–Control Study from Kazakhstan

**DOI:** 10.3390/ijerph23080959

**Published:** 2026-07-25

**Authors:** Togzhan Serbatyrova, Gulnar Shalgumbayeva, Assel Tukinova, Gulnara Sarsebayeva, Laura Danyarova

**Affiliations:** 1 Department of Public Health, NJSC “Semey Medical University”, Semey 071400, Kazakhstan; togjan-2912@mail.ru; 2 Department of General Medical Practice, NJSC “Semey Medical University”, Semey 071400, Kazakhstan; gulnar.shalgumbayeva@smu.edu.kz; 3 Department of Epidemiology and Biostatistics, NJSC “Semey Medical University”, Semey 071400, Kazakhstan; 4 Department of Therapy, NJSC “Semey Medical University”, Semey 071400, Kazakhstan; sarsebaeva2012@mail.ru; 5 Research Institute of Cardiology and Internal Diseases, Almaty 050000, Kazakhstan; lbdanyarova@mail.ru

**Keywords:** gestational diabetes mellitus, pregnancy, obesity, body mass index, hypertensive disorders, macrosomia, case–control study, Kazakhstan

## Abstract

**Highlights:**

**Public health relevance—How does this work relate to a public health issue?**

- Gestational diabetes mellitus is an increasingly important public health concern due to its rising prevalence and its association with adverse maternal and pregnancy outcomes.
- Evidence on factors associated with gestational diabetes mellitus in Central Asian populations remains limited, particularly in Kazakhstan.

**Public health significance—Why is this work of significance to public health?**

- The study identified pre-pregnancy obesity and educational level as factors independently associated with gestational diabetes mellitus.
- Women with gestational diabetes mellitus experienced higher frequencies of hypertensive complications, cesarean delivery, and fetal macrosomia compared with women with normal glucose tolerance.

**Public health implications—What are the key implications or messages for practitioners, policy makers and/or researchers in public health?**

- Strategies aimed at preventing obesity before pregnancy may contribute to reducing the burden of gestational diabetes mellitus and its associated complications.
- Further multicenter prospective studies are needed to improve understanding of gestational diabetes mellitus and support evidence-based maternal health policies in Kazakhstan and other Central Asian countries.

**Abstract:**

Background/Objectives: Gestational diabetes mellitus (GDM) is a common metabolic complication of pregnancy associated with adverse maternal and neonatal outcomes. Data on GDM in Central Asian populations remain limited. This study aimed to identify factors associated with GDM and evaluate selected obstetric outcomes among pregnant women in Kazakhstan. Methods: A retrospective case–control study based on medical record data was conducted among 256 pregnant women, including 106 women with GDM and 150 women with normal glucose tolerance. GDM was diagnosed using a 75 g oral glucose tolerance test according to the International Association of Diabetes and Pregnancy Study Groups criteria. Demographic, clinical, anthropometric, laboratory, and obstetric data were obtained from medical records. Multivariable logistic regression was used to identify factors statistically associated with GDM. Obstetric outcomes were compared using unadjusted analyses. Results: Women with GDM were older than controls (31.6 ± 5.8 vs. 28.6 ± 5.3 years, *p* < 0.001) and had a higher pre-pregnancy body mass index (BMI) (29 [25–33] vs. 26 [23–29] kg/m^2^, *p* < 0.001). Thyroid-stimulating hormone levels were also higher in the GDM group (*p* = 0.026). In multivariable analysis, pre-pregnancy obesity (adjusted OR 4.54, 95% CI 2.08–9.91; *p* < 0.001) and educational level (overall *p* = 0.001) remained associated with GDM, whereas maternal age was not independently associated after adjustment. Hypertension (24.5% vs. 2.0%; *p* < 0.001), eclampsia (3.8% vs. 0%; *p* = 0.028), cesarean delivery (52.8% vs. 28.0%; *p* < 0.001), and fetal macrosomia (21.7% vs. 12.0%; *p* = 0.037) were more frequent among women with GDM. No significant difference was observed in neonatal birth weight. Conclusions: Pre-pregnancy obesity and educational level were independently associated with GDM. Women with GDM more frequently experienced hypertensive complications, cesarean delivery, and fetal macrosomia; however, these findings were based on unadjusted analyses and should be interpreted cautiously. Further multicenter prospective studies are needed to confirm these findings in Central Asian populations.

## 1. Introduction

Gestational diabetes mellitus (GDM) is a condition characterized by elevated blood glucose levels first detected during pregnancy in women with no prior history of diabetes. This disturbance of carbohydrate metabolism develops as a result of pregnancy-related hormonal changes and the increased metabolic demands placed on the maternal organism. GDM is considered one of the most common medical complications in obstetric practice, as it may affect both the course of pregnancy and fetal outcomes, thereby requiring timely monitoring and appropriate management [1,2]. Recent studies indicate that gestational diabetes mellitus is becoming increasingly prevalent worldwide. This rise is associated with several factors, including advanced maternal age at conception, a growing prevalence of obesity, and changes in lifestyle and behavioral patterns, as well as the implementation of updated diagnostic criteria that enable earlier and more accurate detection of the condition [3,4,5]. According to the IDF Diabetes Atlas, the global age-standardized prevalence of gestational diabetes mellitus is approximately 14% (95% confidence interval: 13.97–14.04%). In European countries, this rate ranges from 7.8% to 10.9%, depending on the specific country and the diagnostic criteria applied. In some Eastern European countries, the prevalence of GDM may reach as high as 31.5% [3,4,6]. Analysis of data from the national “Diabetes” registry and the “Registry of Pregnant Women and Women of Reproductive Age” indicates that the prevalence of gestational diabetes mellitus varies across different regions of Kazakhstan, ranging from 4.0% to 11.0%. Notably, in 2021 alone, the number of GDM cases increased by approximately 50% compared to 2020. Over the past five years, there has been a steady rise in type 2 diabetes (T2D) incidence, with women accounting for 65.5% of all patients. The number of new T2D cases increased by 140,000 (46%), of which 85,000 (41%) were women. Among women of reproductive age, the incidence of T2D rose by 58.3%, significantly higher than the overall average for all women, which was 41% [7].

The key factors that increase the risk of gestational diabetes mellitus include maternal age over 30 years, pre-pregnancy overweight or obesity, a family history of type 2 diabetes, previous occurrences of GDM, and belonging to certain ethnic groups. According to research data, women with obesity face a 6.8-fold higher risk of developing GDM compared to women of normal weight, while for women over 30 years of age, the risk increases by approximately 2.1 times [3,8]. In recent years, the molecular and genetic mechanisms underlying the development of gestational diabetes mellitus have also been actively studied, with particular attention given to the roles of adipokines and genetic predisposition in the pathogenesis of the condition [9,10,11]. In Kazakhstan, more than half of pregnant women with gestational diabetes mellitus have abdominal obesity, with the highest prevalence observed in the Almaty region and the lowest in the Aktobe region. Arterial hypertension is also relatively common among women with GDM, although its prevalence is lower in the Aktobe region. The highest proportion of smoking women with GDM has been recorded in Almaty city and the Almaty region [7]. A genomic study of 1800 apparently healthy individuals of Kazakh ethnicity revealed a high frequency of unfavorable variants of the glucokinase gene rs4607517: the A allele was present in 17.6% of participants, the heterozygous A/G genotype in 29.5%, and the homozygous A/A genotype in 3.0%. These findings suggest a potential involvement of hereditary forms of MODY2 in the development of gestational diabetes mellitus among Kazakhs [12].

Gestational diabetes mellitus is associated with an increased risk of various complications for both the mother and the child. These include fetal macrosomia, the need for cesarean delivery, hypertensive disorders, and preterm birth, as well as long-term metabolic disturbances in both mother and child. According to research, women with GDM have a 1.7-fold higher likelihood of delivering a large-for-gestational-age infant, a 1.16-fold higher risk of requiring a cesarean section, and a 1.5-fold increased risk of preterm birth compared to women without GDM [13,14]. In addition, gestational diabetes mellitus increases the likelihood that the mother will develop type 2 diabetes in the future, while the child may experience various metabolic disturbances [1,5]. A cohort study conducted in Astana included 61 pregnant women with gestational diabetes mellitus and 39 women with normal glucose tolerance. Among the GDM patients, the mean pre-pregnancy body mass index was 31.1 ± 7.4 kg/m^2^. The incidence of gestational hypertension reached 36.1% and preeclampsia 14.8%. Uteroplacental and fetoplacental blood flow abnormalities were significantly more frequent (RR = 6.39), as was diabetic fetopathy (RR = 5.12), compared to the control group. In addition, women with GDM experienced higher rates of preterm birth, labor induction, and birth-related injuries [15].

Although knowledge of the risk factors, pathophysiological mechanisms, and consequences of gestational diabetes mellitus has significantly expanded in recent years, there remains a need to improve methods for early detection, accurately assess individual risk, and optimize the management strategies for pregnant women with this condition [5,16,17]. Therefore, the investigation of risk factors and obstetric outcomes in gestational diabetes mellitus plays a key role in reducing disease severity, improving maternal and neonatal prognoses, and developing effective approaches for prevention and timely intervention. Gestational diabetes mellitus poses a significant burden on healthcare systems due to its association with maternal and neonatal complications, increased healthcare costs, and long-term metabolic risks for both mother and child [5]. Despite growing global evidence, there is a lack of epidemiological data on GDM risk factors and outcomes in Central Asian populations, including Kazakhstan.

## 2. Materials and Methods

### 2.1. Study Design and Participants

A retrospective case–control study based on medical record data was conducted at the University Hospital of Semey Medical University, Kazakhstan. Medical records of pregnant women who received antenatal care and delivered at the study center between January 2023 and December 2024 were reviewed.

A total of 256 women were included in the analysis. The case group consisted of 106 women diagnosed with gestational diabetes mellitus (GDM). The control group comprised 150 women with normal glucose tolerance identified from the same hospital and study period. Controls were selected from pregnancies without documented GDM and without major pregnancy complications requiring exclusion from routine antenatal care. Information regarding family history of diabetes, previous GDM, smoking status, and obstetric history was obtained from medical records.

Women aged 18 years or older with singleton pregnancies and available oral glucose tolerance test (OGTT) results were eligible for inclusion. Exclusion criteria included pre-existing type 1 or type 2 diabetes mellitus, multiple pregnancy, severe chronic diseases affecting glucose metabolism, and incomplete medical records.

The study was conducted in accordance with the Declaration of Helsinki and approved by the Local Ethics Committee of Semey Medical University (Protocol No. 2/2023). Written informed consent was obtained from all participants prior to enrollment. All data were anonymized before analysis.

### 2.2. Diagnosis of Gestational Diabetes Mellitus

GDM screening was performed using a 75-g oral glucose tolerance test between 24 and 28 weeks of gestation as part of routine antenatal care.

The diagnosis of GDM was established according to national clinical recommendations based on the International Association of Diabetes and Pregnancy Study Groups (IADPSG) criteria. GDM was diagnosed when at least one of the following plasma glucose thresholds was met or exceeded: fasting glucose ≥ 5.1 mmol/L, 1 h glucose ≥ 10.0 mmol/L, or 2 h glucose ≥ 8.5 mmol/L. Women with normal glucose values at all OGTT time points were included in the control group.

### 2.3. Data Collection

Demographic, clinical, laboratory, and obstetric data were extracted from electronic medical records.

The following variables were collected: maternal age, educational level, place of residence, smoking status, parity, number of previous pregnancies, history of fetal macrosomia, conception by assisted reproductive technologies (ART), family history of diabetes mellitus, previous history of GDM, thyroid-stimulating hormone (TSH), insulin concentration, and pre-pregnancy body mass index (BMI).

Pre-pregnancy BMI was calculated using weight recorded before pregnancy or during the first antenatal visit. BMI was categorized according to World Health Organization criteria as normal weight (18.5–24.9 kg/m^2^), overweight (25.0–29.9 kg/m^2^), and obesity (≥30.0 kg/m^2^).

### 2.4. Obstetric Outcomes

The obstetric outcomes evaluated in this study included hypertensive disorders of pregnancy, eclampsia, cesarean delivery, fetal macrosomia, and neonatal birth weight.

Hypertensive disorders were defined according to diagnoses documented in the medical records. Fetal macrosomia was defined as a birth weight greater than 4000 g.

### 2.5. Statistical Analysis

Statistical analyses were performed using SPSS software version 20.0 (IBM Corp., Armonk, NY, USA).

Continuous variables were assessed for normality using the Shapiro–Wilk test. Variables with normal distribution are presented as mean ± standard deviation (SD), whereas non-normally distributed variables are reported as median and interquartile range (IQR). Categorical variables are expressed as frequencies and percentages.

Comparisons between groups were performed using Student’s *t*-test or the Mann–Whitney U test for continuous variables and Pearson’s chi-square test or Fisher’s exact test for categorical variables, depending on expected cell frequencies. Fisher’s exact test was applied for categorical comparisons with small expected counts or zero cells, including eclampsia, family history of diabetes, and previous history of GDM.

To identify factors statistically associated with GDM, multivariable logistic regression analysis was performed. Variables entered into the model were selected based on clinical relevance and findings from univariable analyses. Adjusted odds ratios (ORs) and 95% confidence intervals (CIs) were calculated.

Available-case analysis was used for variables with missing observations. The number of observations included in each analysis is reported in the corresponding tables.

All statistical tests were two-sided, and *p*-values <0.05 were considered statistically significant.

## 3. Results

A total of 256 pregnant women were included in the study: 106 with gestational diabetes mellitus (GDM) and 150 women in the control group (Table 1).

Women with GDM were significantly older than controls (31.6 ± 5.8 vs. 28.6 ± 5.3 years, *p* < 0.001) and had a higher pre-pregnancy body mass index (BMI) (29 [25–33] vs. 26 [23–29] kg/m^2^, *p* < 0.001). Significant differences were also observed in educational level (*p* = 0.001). Smoking during pregnancy was uncommon in both groups and did not differ significantly (*p* = 0.070). A family history of diabetes mellitus and a history of GDM in previous pregnancies were observed only among women with GDM (*p* < 0.001 and *p* = 0.006, respectively). The proportion of pregnancies conceived using in vitro fertilization was similar in both groups (*p* = 0.700). No significant difference was found in parity between women with GDM and controls (78.3% vs. 74.0% having at least one previous birth, *p* = 0.429). Median thyroid-stimulating hormone (TSH) levels were higher in the GDM group (1.7 [1.02–2.54] vs. 1.3 [1.01–1.93] mIU/L, *p* = 0.026), whereas insulin levels did not differ significantly between groups (*p* = 0.410).

The following variables were included in the multivariable logistic regression analysis: maternal age group, marital status, education level, place of residence, history of macrosomia in previous pregnancies, pregnancy conceived using assisted reproductive technologies (ART), number of pregnancies excluding the current pregnancy, number of previous births, and pre-pregnancy BMI (Table 2).

In the adjusted model, educational level and pre-pregnancy BMI remained associated with GDM. Obesity before pregnancy was associated with higher odds of GDM (adjusted OR 4.54; 95% CI 2.08–9.91; *p* < 0.001). In contrast, maternal age group and history of macrosomia in previous pregnancies were not independently associated with GDM after adjustment. Although educational level showed an overall statistically significant association with GDM (*p* = 0.001), the confidence intervals for individual educational categories were wide and should be interpreted with caution.

Obstetric outcomes are presented in Table 3.

Eclampsia was observed in 4 (3.8%) women with GDM, whereas no cases were recorded in the control group (*p* = 0.028). Hypertension was significantly more frequent among women with GDM than among controls (26 [24.5%] vs. 3 [2.0%], *p* < 0.001). Cesarean section was performed in 56 (52.8%) women with GDM and in 42 (28.0%) women in the control group (*p* < 0.001). Fetal macrosomia (>4000 g) was observed in 23 (21.7%) newborns in the GDM group and in 18 (12.0%) newborns in the control group (*p* = 0.037). Median neonatal birth weight was 3200 (2700–3680) g in the GDM group and 3450 (3200–3700) g in the control group; the difference was not statistically significant (*p* = 0.114).

Because obstetric outcomes were compared using unadjusted analyses, these findings should be interpreted as exploratory associations rather than evidence of causal relationships.

## 4. Discussion

This case–control study investigated factors associated with gestational diabetes mellitus (GDM) and selected obstetric outcomes among pregnant women in Kazakhstan. In unadjusted analyses, women with GDM were older, had a higher pre-pregnancy body mass index (BMI), and had higher thyroid-stimulating hormone (TSH) levels than women with normal glucose tolerance. However, in multivariable logistic regression analysis, only pre-pregnancy obesity and educational level remained significantly associated with GDM, whereas maternal age was no longer independently associated with the condition after adjustment for potential confounders.

The association between pre-pregnancy obesity and GDM observed in our study is consistent with extensive evidence from previous investigations conducted in different populations [18,19,20,21,22,23,24]. Obesity contributes to insulin resistance through multiple metabolic and inflammatory pathways and is considered one of the strongest modifiable factors associated with GDM development [8]. In our study, women with obesity before pregnancy had more than fourfold higher odds of GDM compared with women of normal weight. Similar associations have been reported in studies from Europe, Asia, and North America [18,19,20,21,22,23,24,25]. These findings emphasize the importance of preconception weight management and lifestyle interventions aimed at reducing excess body weight before pregnancy.

Women with GDM were older than controls in the descriptive analysis. However, maternal age was not independently associated with GDM after adjustment in the multivariable model. This finding suggests that the observed age difference may be partially explained by other correlated characteristics, including body weight and reproductive history. Therefore, although advanced maternal age has been reported as an important factor associated with GDM in previous studies [2,18,19,20,21], our findings do not support an independent association within the studied population.

Educational level remained statistically associated with GDM in the adjusted model. Nevertheless, the confidence intervals for individual educational categories were wide and included the null value, indicating substantial statistical uncertainty. Therefore, this finding should be interpreted cautiously. Educational level may reflect broader socioeconomic and behavioral factors that were not directly measured in the present study, including health literacy, dietary patterns, access to healthcare, and health-seeking behavior. Further studies with larger sample sizes are required to clarify this association.

Higher TSH levels were observed among women with GDM in unadjusted analyses. However, TSH was not evaluated as an independent predictor in the final multivariable model, and the median values remained within the laboratory reference range. Consequently, the clinical significance of this finding remains uncertain and should not be interpreted as evidence of an independent relationship between thyroid function and GDM. Additional prospective studies are needed to further explore this potential association.

Regarding obstetric outcomes, hypertensive disorders and eclampsia were more frequently observed among women with GDM. Previous studies and meta-analyses have similarly reported increased frequencies of hypertensive complications among women with GDM [2,19,20,24,26,27,28]. Shared pathophysiological mechanisms, including endothelial dysfunction, chronic inflammation, and insulin resistance, may contribute to the coexistence of these conditions. However, because obstetric outcomes in the present study were evaluated using unadjusted analyses, these findings should be considered exploratory associations and not evidence of causal relationships. Residual confounding by maternal characteristics and other clinical factors cannot be excluded.

Unlike several previous reports [2,19,20,21,22,23,24,29,30], we did not identify clear evidence that GDM was independently associated with adverse neonatal outcomes. Although differences in fetal macrosomia and cesarean delivery were observed between groups, these analyses were not adjusted for potential confounders and therefore should be interpreted cautiously. Furthermore, information regarding gestational age at delivery and large-for-gestational-age status was unavailable, limiting interpretation of neonatal birth weight findings. The relatively lower median birth weight observed in the GDM group may also reflect differences in clinical management during pregnancy.

From a public health perspective, the increasing prevalence of GDM represents an important challenge because of its association with maternal complications, future type 2 diabetes, and increased healthcare utilization [5]. Previous studies have demonstrated that diabetes and its complications impose substantial economic burdens on healthcare systems through increased antenatal monitoring, pharmacological treatment, hospitalization, and long-term follow-up requirements [5]. Our findings support the importance of identifying women with obesity before pregnancy, although larger multicenter studies are needed before broader population-level recommendations can be made.

Several limitations should be considered. First, the case–control design does not permit causal inference and allows identification only of factors associated with GDM. Because controls were selected from a single hospital population, potential selection and ascertainment bias cannot be completely excluded. Differences observed in some baseline characteristics may partly reflect characteristics of the selected control population rather than true population-level differences. Second, this was a single-center study with a relatively modest sample size, which may limit generalizability to other regions of Kazakhstan. Third, some variables contained small cell counts, and several clinically important characteristics, including family history of diabetes and previous GDM, were present only among women with GDM, limiting their statistical evaluation. Fourth, information regarding dietary treatment, insulin therapy, glycemic control during pregnancy, gestational weight gain, and gestational age at delivery was unavailable and therefore could not be incorporated into the analyses. Finally, obstetric outcomes were assessed using unadjusted comparisons and should be interpreted as exploratory findings requiring confirmation in larger prospective studies. This was a retrospective case–control study based on medical record data. Therefore, information on some variables was limited or unavailable, including detailed clinical management during pregnancy, lifestyle factors, and certain longitudinal measurements. Data were extracted exclusively from existing hospital records.

Despite these limitations, this study provides additional evidence regarding factors associated with GDM in a Central Asian population, a region for which epidemiological data remain limited. The findings contribute to the growing body of literature on GDM and may help guide future multicenter investigations aimed at improving maternal health outcomes in Kazakhstan.

## 5. Conclusions

The findings of this study indicate that pre-pregnancy obesity and educational level were associated with gestational diabetes mellitus among pregnant women included in the study population. Women with GDM were older than controls; however, maternal age did not remain significant after adjustment for other variables.

Higher frequencies of hypertensive complications were observed among women with GDM. Nevertheless, these outcome analyses were exploratory and were not adjusted for potential confounding factors. Therefore, the observed differences should be interpreted with caution.

Given the growing burden of GDM and its potential impact on maternal health, further multicenter prospective studies are warranted to better characterize factors associated with GDM and pregnancy outcomes in Central Asian populations.

## Figures and Tables

**Table 1:** Characteristics of pregnant women with gestational diabetes and the control group.

Variable	GDM (*n* = 106)	Control (*n* = 150)	*p*
Age, years	31.6 ± 5.8	28.6 ± 5.3	<0.001
BMI before pregnancy, kg/m^2^	29 (25–33)	26 (23–29)	<0.001
Education, *n* (%)			0.001
*≤Secondary*	7 (6.6)	33 (22.0)	
*Secondary specialized (college)*	38 (35.8)	27 (18.0)	
*Higher*	61 (57.5)	90 (60.0)	
Smoking, *n* (%)			0.07
*Does not smoke*	103 (97.2)	150 (100.0)	
*Smokes/quit during pregnancy*	3 (2.8)	0	
Family history of diabetes (first-degree relatives (parents, brothers, sisters) had diabetes or GDM), *n* (%)			<0.001
*No*	95 (89.6)	150 (100.0)	
*Yes*	11 (10.4)	0	
History of GDM (elevated blood glucose levels observed in previous pregnancies), *n* (%)			0.006
*No*	99 (93.4)	150 (100.0)	
*Yes*	7 (6.6)	0	
Pregnancy after IVF, *n* (%)			0.70
*No*	103 (97.2)	147 (98.0)	
*Yes*	3 (2.8)	3 (2.0)	
Number of births, *n* (%)			0.429
≥1	83 (78.3)	111 (74.0)	
0	23 (21.7)	39 (26.0)	
TSH, mIU/L	1.7 (1.02–2.54)	1.3 (1.01–1.93)	0.026
Insulin, µIU/mL	29.7 (10.6–69.5)	34.8 (11.8–79.6)	0.41

**Table 2:** Multivariable logistic regression analysis of factors associated with GDM.

Variable	Unadjusted OR	(95% CI)	*p*-Value	Adjusted OR	(95% CI)	*p*-Value
**Age group (years)**			0.027			0.971
Up to 25	1	Reference		1	Reference	
26–35	0.35	0.159–0.751		1.09	0.539–2.188	
≥36	0.55	0.282–1.057		1.03	0.409–2.611	
**Marital status**			0.304			
Married	1	Reference				
Single	1.76	0.600–5.143				
**Education**			0.001			0.001
Higher	1	Reference		1	Reference	
Secondary vocational (technical)	0.77	0.073–8.193		0.63	0.053–7.486	
Secondary	5.43	0.575–51.249		5.32	0.508–55.777	
Uncompleted secondary	2.81	0.306–25.710		3.31	0.323–33.896	
**Residence**			0.369			
Urban	0.78	0.449–1.347				
Rural	1	Reference				
**History of macrosomia in previous pregnancies (birth weight > 4000 g)**			0.040			0.217
Yes	0.49	0.251–0.967		1.67	0.739–3.780	
No	1	Reference		1	Reference	
Pregnancy conceived using ART *			0.685			
Yes	0.72	0.142–3.615				
No	1	Reference				
**Number of pregnancies, excluding the current pregnancy**			0.811			
0	0.67	0.279–1.612				
1–2	0.71	0.325–1.531				
3–4	0.76	0.336–1.703				
≥5	1	Reference				
**Number of births**			0.984			
0	1	Reference				
1–2	1.01	0.515–1.989				
3–4	1.13	0.537–2.367				
≥5	-	-				
**BMI before pregnancy**			0.000			0.000
Normal	1	Reference				
Overweight	0.96	0.525–1.756		0.76	0.401–1.442	
Obesity	4.91	2.483–9.725		4.54	2.080–9.911	

* ART—assisted reproductive technologies.

**Table 3:** Obstetric outcomes in women with gestational diabetes mellitus (GDM) and in the control group.

Variable	GDM	Control	*p*
Eclampsia			0.028
*no*	102 (96.2)	150 (100.0)	
*yes*	4 (3.8)	0	
Hypertension			<0.001
*no*	80 (75.5)	147 (98.0)	
*yes*	26 (24.5)	3 (2.0)	
Cesarean section			<0.001
*no*	50 (47.2)	108 (72.0)	
*yes*	56 (52.8)	42 (28.0)	
Macrosomia			0.037
*<4000 g*	83 (78.3)	132 (88.0)	
≥4000 g	23 (21.7)	18 (12.0)	
Neonatal birth weight, g	3200 (2700–3680)	3450 (3200–3700)	0.114

## Data Availability

All data generated or analyzed during this study are included in this published article.
